# An Inflammatory Pseudotumor Arising from Pterygopalatine Fossa with Invasion to the Maxillary Sinus and Orbital Cavity

**DOI:** 10.1155/2015/950823

**Published:** 2015-06-18

**Authors:** Hidenori Yokoi, Takuya Yazawa, Yuma Matsumoto, Tetsuya Ikeda, Masachika Fujiwara, Yasuo Ohkura, Naoyuki Kohno

**Affiliations:** ^1^Department of Otolaryngology, Head and Neck Surgery, Kyorin University School of Medicine, 6-20-2 Shinkawa, Mitaka, Tokyo 181-8611, Japan; ^2^Department of Pathology, Kyorin University School of Medicine, 6-20-2 Shinkawa, Mitaka, Tokyo 181-8611, Japan

## Abstract

We report a patient who had an inflammatory pseudotumor (IPT) that invaded to the maxillary sinus and orbital cavity, with the left pterygopalatine fossa as the principal site; this is a very rare case. The patient was an 83-year-old woman who suddenly became aware of impairment in the eyesight and visual field of the left eye. CT images showed a neoplastic lesion that invaded to the maxillary sinus and orbital cavity, with the left pterygopalatine fossa as the principal site, and also showed contrast effects. To obtain a definitive diagnosis from histopathological analysis, the lesion was biopsied, and she was diagnosed as the inflammatory pseudotumor with the immunohistochemical study and multiplex polymerase chain reaction-based clonality assays. The patient had a lymphoid-predominant lesion that responded to radiotherapy but corticosteroids were not effective. It is important to scrutinize the pathology to avoid unnecessary and mutilating surgery.

## 1. Introduction

An inflammatory pseudotumor (IPT) is a space-occupying lesion that causes neoplastic proliferation clinically but is a histopathologically benign disease involving nonspecific chronic inflammation [1].

IPTs are commonly found in the lungs [2] and inside the orbital cavity [3] and are difficult to diagnose because clinically IPT is similar to malignant disease [4]. Here, we report a rare case of IPT with the left pterygopalatine fossa as the principal site, which invaded the maxillary sinus and orbital cavity. In addition, we reviewed pertinent recent literature and included discussions on the findings from these literatures.

## 2. Case

An 83-year-old woman suddenly became aware of impairment in her eyesight and visual field of the left eye. She was admitted to the ophthalmology department in our hospital because she did not experience any improvement with observation. During an eyesight examination, abnormal vision was confirmed in her left eye.

The patient had a history of lung cancer for which she had undergone surgery 10 years ago and arrhythmia for which she had a pacemaker implanted 5 years ago.

**Figure 1:**
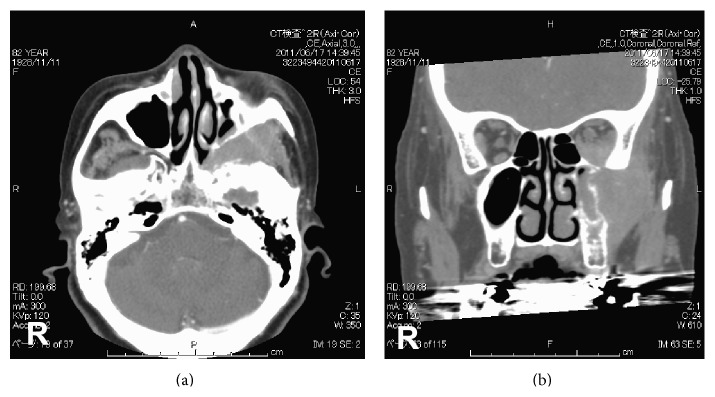
A neoplastic lesion with contrast effects and an indistinct boundary, which had invaded to the maxillary sinus and orbital cavity. The principal site of the lesion was the left pterygopalatine fossa.

Computed tomography (CT) images showed a neoplastic lesion with contrast enhancement and an indistinct boundary, which had invaded to the maxillary sinus and orbital cavity. The principal site of the lesion was the left pterygopalatine fossa. In addition, the images also showed bone destruction of the lateral wall of the maxillary sinus because of exclusion and invasion of the lesion at its outer side (Figure 1). Magnetic resonance imaging (MRI) could not be performed because the patient had a pacemaker.

Eyesight examination test showed that the best corrected visual acuity (BCVA) of the left eye was 0.2. The visual field test showed enlargement of the blind spot. The soluble interleukin-2 receptor (sIL-2R) level was a little high at 576 U/mL (normal range: 145 U/mL–519 U/mL); however, abnormalities pertaining to inflammation markers, tumor markers, and collagen disease markers were not noted during another blood test.

We performed a biopsy of the lesion to obtain a definitive histopathological diagnosis. First, the uncinate process was removed, and then the maxillary ostium was opened. The tumor mass was removed from a part of the maxillary sinus and the pterygopalatine fossa.

**Figure 2:**
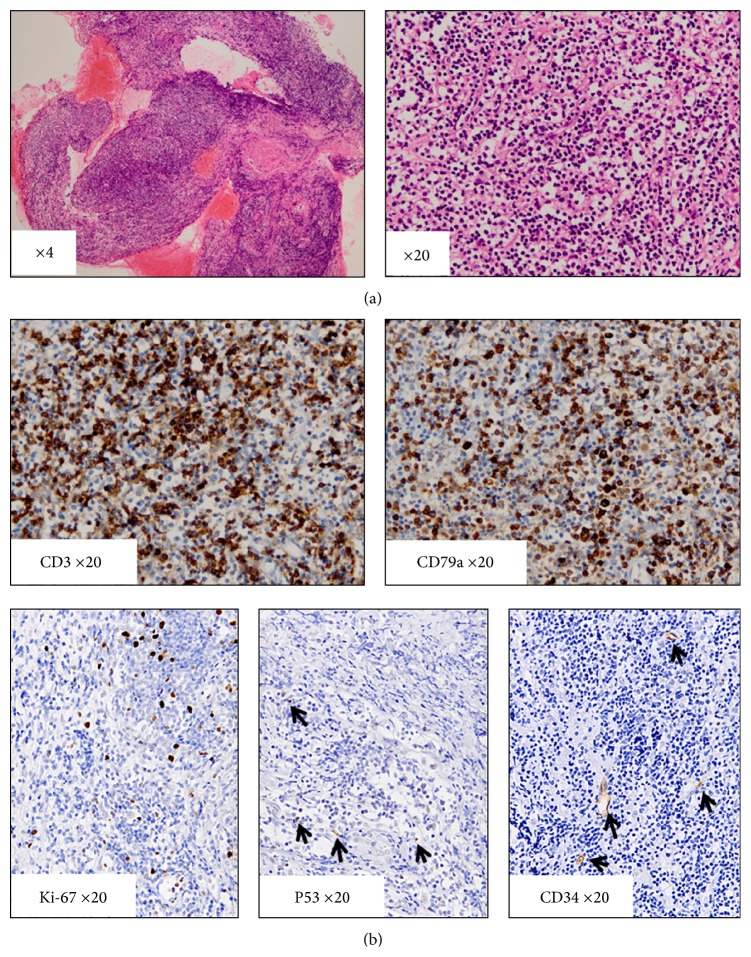
(a) Hematoxylin-eosin (HE) staining. Marked lymphocytic infiltration was found in the lesion. Infiltrating lymphocytes were small in size. (b) Immunohistochemical staining. Infiltrating lymphocytes consisted of CD3-positive T-cells and CD79a-positive B-cells. Infiltrating lymphocytes revealed low Ki-67 and P53 positivity. CD34 expression was limited in the vascular endothelial cells.

Hematoxylin-eosin (HE) staining of the biopsied specimen showed dense infiltration of small lymphocytes, which possessed equally sized round nuclei with a fine chromatin pattern. There was also an admixture of small numbers of plasma cells and eosinophils. The lymphocytic infiltration did not reveal nodularity or lymphoepithelial lesions of the sinonasal gland (Figure 2(a)). Immunohistochemistry showed both CD3-positive T-cells and CD79a-positive B-cells infiltrated to the lesion (Figure 2(b)). Among the plasma cell, there was no predomination for kappa- or lambda-positive ones. IgG4-positive plasma cells were scarcely encountered. Proliferation of ALK-positive myofibroblasts or CD21-positive follicular dendritic cells was not demonstrated. In situ hybridization for EBV-encoded RNA (EBER) gave negative results. Additional immunohistochemical analysis was performed to analyze the proliferative capacity of the lesion using Ki-67 and P53. The Ki-67 labeling index was approximately 10–15%, and P53-positive lymphocytes were hardly seen (Figure 2(b)). Furthermore, positive signals of CD34 were restricted in blood vessels (Figure 2(b)). These data showed mixed infiltration of mature T- and B-cells with a low proliferative capacity.

**Figure 3:**
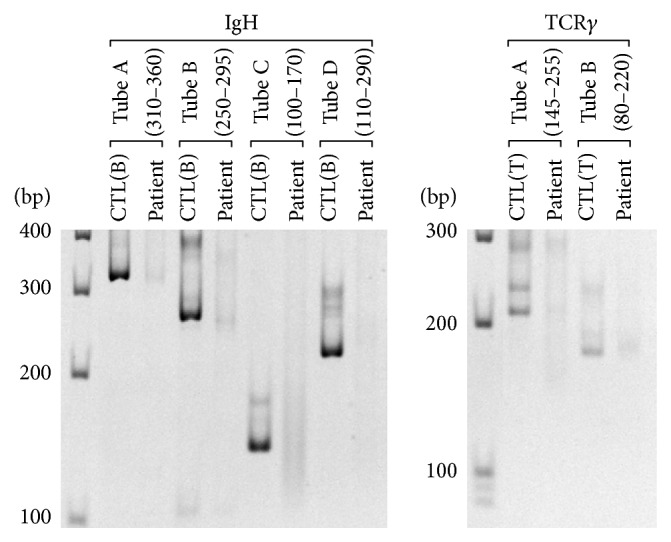
Results of multiplex PCR as to immunoglobulin heavy chain (IgH) and T-cell receptor gamma (TCR*γ*) genes. Multiplex PCR and acrylamide gel electrophoresis were carried out according to the standardized BIOMED-2 PCR protocol. Predicted sizes of PCR products are indicated beneath the tube names. CTL(B): positive control using DNA extracted from B-cell lymphoma cells. CTL(T): positive control using DNA extracted from T-cell lymphoma cells. bp: base pair.

To examine the possibility of lymphoproliferative disorders, multiplex PCR-based clonality assays as to VH-JH region of immunoglobulin heavy chain (IgH) and T-cell receptor gamma (TCR*γ*) were conducted using genomic DNA extracted from the formalin-fixed paraffin-embedded tissue samples and BIOMED-2 primer sets [5]. As shown in Figure 3, definite results suggesting malignant lymphoma were not obtained. From these histological, immunohistochemical, in situ hybridization, and multiplex PCR findings, we diagnosed the lesion as IPT that was not suitable for known neoplastic conditions, such as inflammatory myofibroblastic tumor or EBV-positive inflammatory follicular dendritic cell tumor.

**Figure 4:**
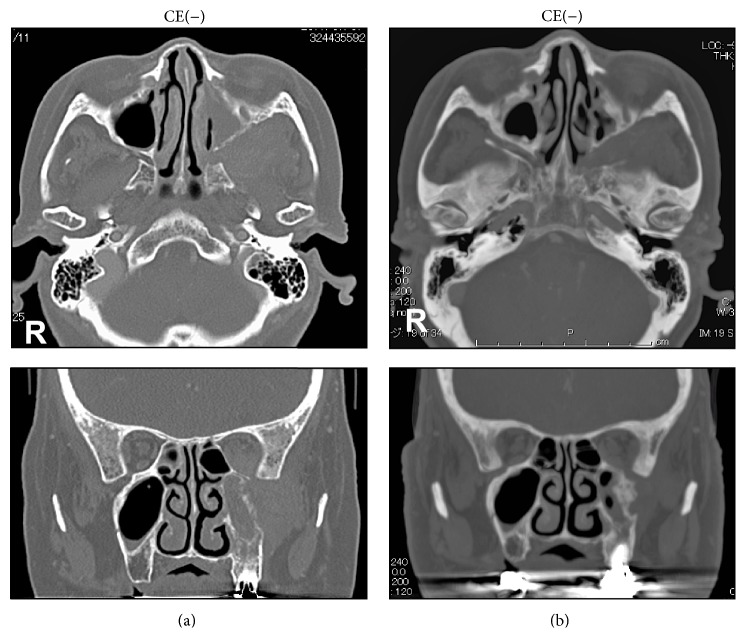
(a) Steroid therapy showed no change in the lesion. (b) Radiotherapy showed almost the entire lesion disappearing.

The clinical course after biopsy included steroid pulse therapy according to the following protocol: 1 g of methylprednisolone sodium succinate for 3 days, followed by prednisolone, for which the dosage was gradually reduced from 40 mg to 10 mg and finally to 5 mg. However, no apparent vision recovery was observed during eyesight examination. Subsequently, we decided to administer radiotherapy. The patient received a total of 40 Gy radiotherapy, which was very effective, and her left eye vision recovered with a BCVA of 0.7.The CT images after steroid therapy showed no change in the lesion (Figure 4(a)); however, after radiotherapy, almost the entire lesion disappeared (Figure 4(b)).

The patient is well and free of disease after the therapy for three years.

## 3. Discussion

IPT was initially reported as a nonneoplastic mass occurring in the lungs [6]. However, there have been reports of its appearance in various different organs, including kidneys, spleen, stomach, and liver [6, 7], and in the head and neck region. The occurrence of IPT has been reported in sites, including the cranium [8], orbital cavity, larynx, tonsils, and thyroid [7, 8]. IPT is also known as plasma cell granuloma, and it is most commonly seen in the lungs [2]. It has been reported that IPTs outside the lungs differ from those within the lungs, and whether all these IPTs actually belong to a single histopathological category called “IPT” is a matter of controversy [9]. Our study patient had undergone lung cancer surgery 10 years ago, and although the histopathological details were unavailable, whether the 2 conditions, lung cancer and IPT, are related is an extremely interesting question.

The nasal sinuses, particularly the maxillary sinus, can be the site of not only carcinomas, such as squamous-cell epithelial carcinoma, but also hematopoietic malignant tumors, such as malignant lymphoma and extramedullary plasmacytoma, and autoimmune diseases, such as Wegener's granuloma, making histopathological differential diagnosis problematic.

Although pseudotumor with the pterygopalatine fossa as the principal site is rare, a few cases have been reported, and in all these cases, the IPTs were difficult to differentiate from malignant tumors [10], which is similar to that observed in our study. Malignant lymphoma is considered to be the most important as a differential diagnosis. In general, malignant lymphoma is histopathologically characterized by monoclonal proliferation and confirmed by HE staining of monoclonal proliferation of atypical lymphocytes; immunostaining shows the monoclonality of lymphocytes (particularly light-chain immunoglobulins), which is important for diagnosis. In the case of nasal natural killer (NK)/T-cell lymphoma, which frequently occurs in the nasal sinuses, prominent polymorphous proliferation of lymphocytes is present, and that is evident by its former name, polymorphic reticulosis. NK/T-cell lymphoma also exhibits varied histology, including large monocytes and multinucleated cells mixed with plasmacytes and histiocytes, which means differentiation solely by HE staining is problematic. IgG4-related disorder is also considered to be important as a differential diagnosis [11] but HE staining showed almost no plasmacytic infiltration, and the level of serum IgG4 was normal. Furthermore, the result of immunohistochemical study revealed that IgG4 positive plasma cells were scarcely encountered. Therefore, we ruled out the possibility of an IgG4-related disorder in the present case.

The histology results of our study were unlike those of chronic conditions in which epithelioid and giant cells, such as sarcoidosis and tuberculosis, are present. Further, we did not observe necrotic granuloma or vasculitis, thereby, differed from Wegener's granuloma and periarteritis nodosa. It was evident that no tumor cells were present during histological analysis in our case, monoclonality could not be shown by immunostaining, and the proliferative potential of the tumor was not particularly high. Thus, considering the overall clinical findings, we diagnosed the patient as IPT.

In this nonfocal case, CT image showed that the principal site was the left pterygopalatine fossa, causing displacement of the inferior lateral wall bone into the maxillary sinus of that side and its pervasive destruction; further, the tumor had advanced into the orbital floor. The fact that the only subjective symptom was visual impairment suggested that the tumor had invaded the ophthalmological region via soft tissue, which was in turn invaded via the inferior orbital fissure from the pterygopalatine fossa.

Previous studies have shown the effectiveness of therapy, such as high-dose corticosteroids, surgical excision, or radiotherapy, for IPTs [8]. A literature review showed that radiotherapy is proposed for lymphoid-predominant lesions, whereas high-dose corticosteroids may be a better choice for granulomatous lesions [12]. Our patient had a lymphoid-predominant lesion that responded to radiotherapy. Therefore, it is important to assess the pathology to avoid unnecessary and mutilating surgery.

Here, we describe the rare case of a patient having IPT with the left pterygopalatine fossa as the principal site that invaded the maxillary sinus and orbital cavity. Previous reports have shown an association between IPT, chromosomal abnormalities, and P80 and ALK1 expressions [13]. Although immunohistochemical results and clonality assay did not reveal neoplastic findings, long-term monitoring should be required to assess future recurrences.
